# EMD: an ensemble algorithm for discovering regulatory motifs in DNA sequences

**DOI:** 10.1186/1471-2105-7-342

**Published:** 2006-07-13

**Authors:** Jianjun Hu, Yifeng D Yang, Daisuke Kihara

**Affiliations:** 1 Department of Computer Science, Purdue University, West Lafayette, IN, 47907, USA; 2 Department of Biological Sciences, Purdue University, West Lafayette, IN, 47907, USA; 3 Markey Center for Structural Biology, Purdue University, West Lafayette, IN, 47907, USA; 4 The Bindley Bioscience Center, Discovery Park, Purdue University, West Lafayette, IN, 47907, USA

## Abstract

**Background:**

Understanding gene regulatory networks has become one of the central research problems in bioinformatics. More than thirty algorithms have been proposed to identify DNA regulatory sites during the past thirty years. However, the prediction accuracy of these algorithms is still quite low. Ensemble algorithms have emerged as an effective strategy in bioinformatics for improving the prediction accuracy by exploiting the synergetic prediction capability of multiple algorithms.

**Results:**

We proposed a novel clustering-based ensemble algorithm named EMD for *de novo *motif discovery by combining multiple predictions from multiple runs of one or more base component algorithms. The ensemble approach is applied to the motif discovery problem for the first time. The algorithm is tested on a benchmark dataset generated from *E. coli *RegulonDB. The EMD algorithm has achieved 22.4% improvement in terms of the nucleotide level prediction accuracy over the best stand-alone component algorithm. The advantage of the EMD algorithm is more significant for shorter input sequences, but most importantly, it always outperforms or at least stays at the same performance level of the stand-alone component algorithms even for longer sequences.

**Conclusion:**

We proposed an ensemble approach for the motif discovery problem by taking advantage of the availability of a large number of motif discovery programs. We have shown that the ensemble approach is an effective strategy for improving both sensitivity and specificity, thus the accuracy of the prediction. The advantage of the EMD algorithm is its flexibility in the sense that a new powerful algorithm can be easily added to the system.

## Background

Identifying gene regulatory and gene expression networks has become a central problem of post-genomics biology [1-3]. Computational prediction of DNA regulatory elements or binding sites of transcription factors is one of the essential parts of the problem. This regulatory motif discovery problem has been studied since the early years of bioinformatics, resulting more than thirty algorithms proposed, among which more than a dozen are publicly available [4,5]. However, recent comprehensive evaluations of existing motif discovery programs show that their prediction accuracy is still very low [4,6].

The technical difficulty resides in the low signal/noise ratio of this problem. A straightforward direction to improve the prediction accuracy is to use a better motif model to capture characteristics of sequence patterns of regulatory motifs (*e.g*. position-dependence information can be captured by a Hidden Markov model [7] or by considering local pairwise nucleotide frequency [8], rather than a conventional position specific scoring matrix [9]) and a better search algorithm in the sequence space (*e.g*. Expectation Maximization [10] or Gibbs sampling [11]). Another approach is to incorporate additional information, such as phylogenetic trees or homologous sequences [12-15]. Comparative genomics [16] and gene expression data [17] have also been used to improve the specificity of motif discovery.

Here, we introduce another practical and powerful strategy for the motif discovery problem, that is, the ensemble approach, which is also called the meta-server approach or the jury-method. The fundamental idea of the ensemble approach is to run several different programs (multiple times) and summarize their outputs to generate the final output. Ensemble algorithms have been applied in several prediction methods in bioinformatics, such as gene prediction [18], protein tertiary structure prediction [19,20], protein domain prediction [21] and protein secondary structure prediction [22,23]. The most remarkable success of the ensemble approach would be the several meta-servers which participated in the biennial world-wide protein structure prediction contest, CASP (Critical Assessment of Techniques for Protein Structure Prediction), in 2002 and 2004 [24-27], which dominated the top ranks in the competition. The success of the ensemble approaches has been attributed to several factors. Albrecht et al. [22] referred their success to the noise-filtering properties of the ensemble approach, which damp the training errors of single methods. Lundström *et al*. [28] discussed that a key for the success of an ensemble approach is to properly measure the similarity between the different models. Most ensemble algorithms use the same type of input data to make final predictions. In contrast, the ensemble algorithm by Sen *et al*. [29] combined different types of data, data mining results, threading, phylogenetic tree-based conserved residue prediction, and the structural alignment based prediction method to predict a protein-protein interaction site in a query protein tertiary structure. Despite the wide range of applications of the ensemble approach in bioinformatics, to the best of our knowledge, there is no extensive study of ensemble algorithms for the motif discovery problem.

In our previous work [6], we showed anecdotal evidence that a simple ensemble motif discovery algorithm, called CEA, outperforms single stand-alone algorithms. At this juncture, it would be appropriate to clarify the differences between the previous work and the current work, called the EMD algorithm. In the previous work, 1) only the combination of multiple runs of an identical algorithm was considered; and 2) only one data set with a short sequence size (50 nt. long sequence added to both sides of each of known target site) was used in the benchmark. In this work: 1) We extend our ensemble approach by systematically combining predictions from five popular motif discovery algorithms, namely, AlignACE [30], BioProspector [31], MDScan [32], MEME [33], and MotifSampler [34]. In addition, we integrated Projection [35] to seek further improvement in terms of the scalability of EMD. All the possible combinations of one to five component algorithms are examined. 2) To be able to combine predictions of different runs from different component algorithms, a novel ensemble algorithm, EMD, is developed. 3) EMD is tested on two different types of data sets. One data set is generated from the intergenic regions of the *E. coli *genome, and the other is input sequences of different lengths generated by adding margins of different sizes to each known site. The best ensemble algorithm performed 22.4% better than the best single component algorithm in terms of the nucleotide level accuracy.

## Results

**Figure 1:**
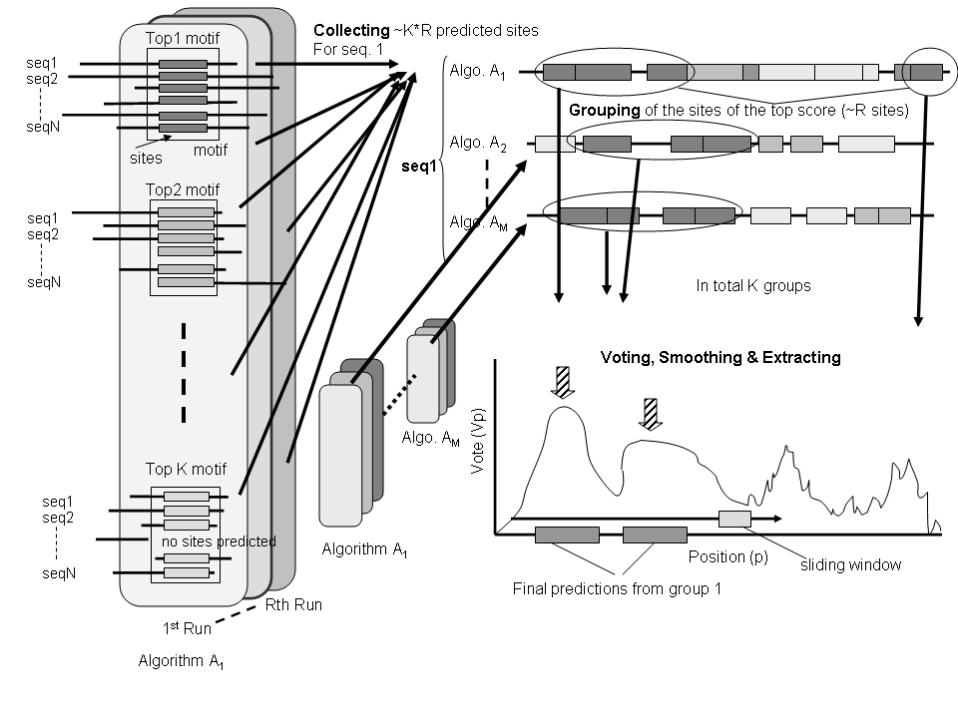
Overview of the EMD algorithm. After each component algorithm is run *R *times for an input sequence data set, *K *motifs are collected from each run. The right side of the figure illustrates the grouping phase of the algorithm for the sequence number 1 and the final prediction of sites for the site group number 1 of the input sequence No. 1. See the text for the details.

We developed a series of the EMD algorithms with all the possible combinations of two to five component algorithms. An EMD algorithm runs its component algorithms multiple times independently, and summarizes their results basically by majority (Fig. 1). The potential of an EMD algorithm lies in the fact that it could take advantage of superb predictions of every component algorithm. The five component algorithms used are AlignACE (AL) [30], BioProspector (BP) [31], MDScan (MD) [32], MEME (ME) [33], and MotifSampler (MS) [34]. Below in the manuscript EMD-X denotes a set of EMD algorithms with all the possible combinations of X number of component algorithms. A multi-restart algorithm and random algorithm are also included as comparison bases. We name different ensemble algorithms by concatenating the abbreviations of the component algorithms: For example, AL-BP is an EMD algorithm with AlignACE (AL) and BioProspector (BP) used as the component algorithms.

### Results of EMD on ECRDB62A intergenic sequence data set

In Table 1, the performance of the best ensemble algorithm in EMD-2 to EMD-5 is compared with that of the five stand-alone component algorithms, five multi-restart algorithms (RS-XX) and a random algorithm on the intergenic sequence data set. The performance is evaluated by the nucleotide level and site level accuracy in terms of the performance coefficient (n/sPC), sensitivity (n/sSn) and specificity (n/sSp). First, the nucleotide level performance coefficient (nPC) is very low for single component algorithms. Even for the best algorithm, BioProspector, nPC does not exceed 0.18. It is remarkable that all of the four ensemble algorithms outperform the best single component algorithm in terms of nPC. EMD-AL-BP-MD is the best ensemble algorithm, achieving an nPC score of 0.213. This is a 22.4% improvement over the best single algorithm, BioProspector, whose nPC is 0.174. Note here that the improvement of EMD over its component algorithms comes from an increase of both the sensitivity and specificity. Surprisingly, the performance of the multi-restart algorithms is worse than that of the corresponding single component algorithm in each case (*e.g*. RS-AL is worse than AlignACE). nPC, nSn and nSp all dropped. This is because the score given to predicted motifs by the algorithms does not always reflect the accuracy well [6] and also collecting the highest-scoring motifs from multiple runs can result in only picking up similar or identical motif predictions and neglecting the sub-optimal motifs. This observation highlights the necessity of reporting multiple top-scored motif predictions rather than reporting a single top predicted motif. The comparison of the EMD algorithms with the multiple-restart algorithms illustrates that the improved performance of the EMD algorithms is not simply due to the increased number of runs of component algorithms but a synergetic effect of the multiple runs.

At the site level, the EMD-AL-BP-MD again achieves the highest prediction performance in sPC, sSn, and sSp. All of the EMD algorithms exceed 0.30 in terms of sPC. The same arguments above for the nucleotide level accuracy also hold for the site level performance.

### Comparison of different combinations of component algorithms

With five component algorithms, there are a total of 31 unique combinations to compose an ensemble algorithm. Table 2 shows the nPC scores for EMD algorithms with all possible combinations of component algorithms tested on ECRDB61B-200 data set (200 nt placed on both sides of known sites). Note that the four EMD algorithms in Table 1 are the best EMD algorithms amongst those with the same number of employed component algorithms benchmarked on ECRDB62A dataset. Please also note that the algorithms shown in Table 2 are all EMD algorithms even if some EMD algorithms only employ a single component algorithm; e.g. EMD-AL in Table 2, results of 20 runs of AlignACE are combined.

Firstly, on average the accuracy improves as the number of component algorithms increases (Table 2). The average accuracy of EMD algorithms with a single component algorithm is 0.202, and the average accuracy monotonically increases up to 0.254 by the EMD algorithm with five component algorithms. Secondly, the standard deviation of the accuracy among the EMD algorithms with the same number of component algorithms is small, which ranges from 0.008 (EMD-4) to 0.015 (EMD-1). These results prove the positive effect of the ensemble approach in the motif discovery problem. The standard deviation of EMD-X algorithms decreases as the number of component algorithms increases. It is observed that MD, which is the best algorithm in constructing EMD algorithms with a single component algorithm, is always involved in the best EMD algorithm among those with a higher number of component algorithms (EMD-2 to EMD-4). Interestingly, AL, which performed the worst in the single component EMD algorithm with nPC of 0.178, can contribute to improved accuracy. Indeed AL-MD-ME performed the best among the EMD-3. These results vividly show the synergetic effect of the EMD algorithm. Note here that the standard error of each EMD algorithm is very small. Indeed it is less than 0.003 for all of the cases, measured from twenty independent runs. This is consistent with our previous observation that the standard deviation of results (nPC) of the single component algorithms is very small [6].

### Number of runs of component algorithms on EMD performance

The number of runs of each component algorithms is an important parameter in an EMD algorithm. It can affect the prediction performance and it also determines the required computational time. We examined the effect of the number of different runs of component algorithms in terms of the nucleotide level prediction accuracy (nPC) (Table 3). In Table 3, the number of runs of the EMD algorithms composed of two component algorithms is changed from 5 to 20 with an interval of 5 runs. More generally, any combinations of different number of runs can be assigned to each component algorithm. However, the exhaustive combinations of different number of runs have not been tried here because the current study would be enough to observe the behavior of EMD algorithms and also because it is too computationally expensive.

From Table 3, it can be seen that on average, increasing the number of runs contributes to the improvement of the performance (see the average nPC value at the bottom row). This general trend does not necessarily apply to a particular EMD algorithm. The optimal number of runs for a particular EMD algorithm may be reflecting characteristics of component algorithms, and not very straightforward to determine. However, the general trend exists that the more number of runs increases the accuracy, and more importantly, the accuracy does not show a dramatic degeneration when the number of runs increases. Therefore, practically for the EMD algorithm, it is not inappropriate to set the number of runs to 20 times or probably any number between 10 and 20.

### Scalability

**Figure 2:**
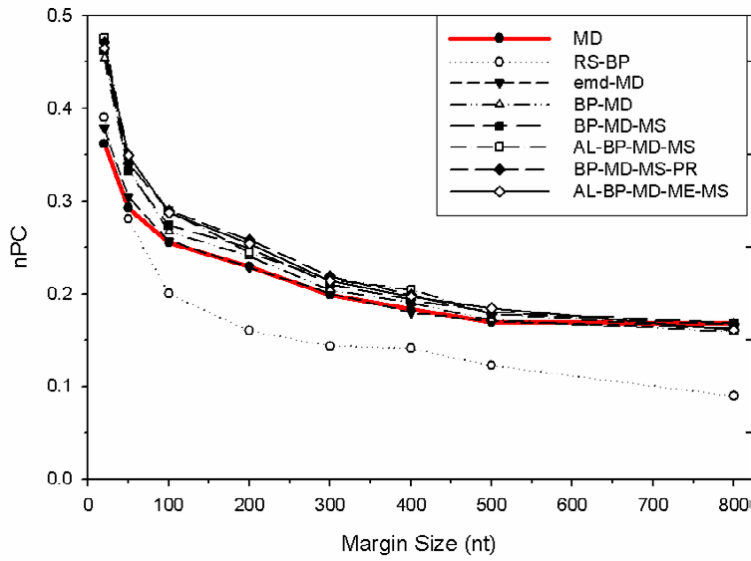
Scalability of the EMD algorithms. The nucleotide level prediction performance was compared with the best base algorithm MDscan (MD) and the best multi-restart algorithm (RS-BP). This evaluation is done on ECRDB61B-200 data set. The y-axis shows the nucleotide level accuracy (nPC). The error bars are not shown because the standard error is very small (less than 0.003).

We examined the scalability of the EMD algorithms in terms of the length of input sequences (Fig. 2). One algorithm from each EMD-X (X ranges from 1 to 5) is examined. Input sequences of different length are prepared as ECRDB61B data set with different margin sizes ranging from 20 to 800 (these correspond to the sequence lengths ranging from approximately 60 to 1620 nt, because the sequence length is the total of two margins and a site width). For comparison, the results of the best individual component algorithm, MDScan (MD), and the best multi-restart algorithm of BioProspector (RS-BP) are also shown.

Figure 2 shows that all of the EMD algorithms outperform MD and RS-BP in terms of nPC when the margin size is up to 200. Note that error bars are not drawn because the standard error of all EMD algorithms is less than 0.003 when computed over twenty runs. The performance of the RS-BP algorithm drops sharply as the margin size increases. This is consistent with the observation from Table 1 that multi-restart algorithms perform the worst. For the margin size of 300 or longer, the performance of all the algorithms start to converge, and the performance of the EMD algorithms does not show a large improvement over single MD algorithm.

One possible reason for the ineffectiveness of EMD algorithms for the data sets with longer sequences may be that only 20 runs of the component algorithms are not sufficient. To check whether increasing the number of runs can improve the results, we run the AL-BP-MD-MS algorithm with different numbers of runs ranging from 10 to 50 with a step size of 10. The corresponding nPC scores were 0.183, 0.187, 0.181, 0.185, and 0.187, respectively. Therefore, there is no significant performance improvement observed when up to fifty runs are conducted for each of the five component algorithms. To ameliorate the loss of advantage for longer margins, we have also added another algorithm, Projection [35], which has a good performance at longer padding sequence length, to EMD-BP-MD-MS algorithm (thus EMD-BP-MD-MS-PR algorithm is composed). The EMD-BP-MD-MS-PR did perform the best for a sequence of the margin size of 100, 200 and 300, but did not show a superior performance for a longer margin size.

At this point, the scalability is still an issue for the future work, but note that the majority of the intergenic regions in *E. coli *are short. Indeed the average length of the intergenic regions of *E. coli *is 300 nt, and 95.2% of them are shorter than 420 nt (which corresponds to the sequences of a margin size of 200), where the EMD algorithms showed their superiority over the component algorithm (Fig 2).

### Performance on the dataset with shuffled margin sequences

We have carried out additional testing on a dataset with artificially shuffled margin sequences, named ECRDB61C (Tab. 4). A drawback of the ECRDB62A and ECRDB61B datasets used above is that some input sequences contain multiple sites, so that the computed accuracy on the datasets may not precisely reflect the actual performance of the algorithms. In contrast, sequences in the ECRDB61C set has a target site in the middle of the sequence with artificially shuffled flanking sequences on both sides, thereby it is guaranteed that only one target site exists in a sequence. We tested AL-BP-MD-ME-MS, because this combination achieved the best nPC on ECRDB61B-200 data set (Tab. 2). The results show that the algorithm performed better on the shuffled margin sequences (ECRDB61C) when the input sequence is short (the margin size of 100), but the performance difference vanishes as the input sequence length increases. The better specificity (nSp) largely contributed in the improvement in the nPC for ECRDB61C-100.

## Discussion

We have developed the EMD algorithm, a framework of an ensemble algorithm for regulatory site motif discovery. The ensemble approach has been successfully applied in several prediction methods in bioinformatics [19,22,28,36]. The importance of comparing results of different programs is also recognized in the field of motif discovery. Melina is a web-based tool which help visualize and compare outputs of various DNA motif finding programs [37]. However, to the best of our knowledge, this is the first extensive report of an ensemble approach for DNA motif discovery, which combines component algorithms to improved regulatory motif predictions. Using the framework we developed, we have tested all the possible combinations of five component algorithms on a benchmark dataset of experimentally verified regulatory motifs in *E. coli*. In terms of the nucleotide level accuracy (nPC) on intergenic region data set, the best EMD algorithm, AL-BP-MD achieved about 4 points (or 22.4%) better accuracy than that of the best component algorithm, BioProspector (Table 1). Considering the low prediction accuracy of current single component algorithms, this improvement is significant. The advantage of the EMD algorithms over the single algorithms decreases for a long input sequence set. However, importantly, the performance (nPC) of the EMD algorithms was never worse than single algorithms, therefore, users of the EMD algorithms will never lose accuracy by using them.

The largest advantage of the EMD algorithm is its flexibility of incorporating new component algorithms. That is, if a novel superior motif discovery algorithm is made available, it can be readily incorporated to the EMD algorithm system. In this study, the five component algorithms we employed were all sequence-based algorithms, because they are easily available on the internet and the basis of more recent algorithms. But there is no difficulty in incorporating advanced motif discovery algorithms which use additional information such as a phylogenetic tree, because the essence of the EMD algorithm is to combine individual independent predictions. Another advantage of the EMD algorithm is that it is particularly suitable for running on a distributed computer system, such as a grid computing or a Linux cluster, which will be certainly one of the main computational powers in the next generation.

Below we discuss major considerations in designing a good ensemble algorithm: 1) Consensus function: how to combine predictions from different algorithms with different confidence levels; 2) Diversity of predictions: how to select and run component algorithms to obtain diverse predictions to ensure successful combination; and 3) Strength of component predictions: ways to weight the predictions from different algorithms.

### Ensemble grouping and weighting

The key of an ensemble approach is how to assemble individual independent predictions from different algorithms. In the current implementation, predicted sites from each algorithm are sorted and grouped first by their score, then further grouped across the results from different algorithms. The intention of this implementation is to increase the specificity of the final prediction by considering the significance of predicted sites. Thus, commonly predicted sites with a high score by different algorithms will be picked up well. In this way, the weight to predicted sites defined by the score is implicitly counted.

For further improvement of the ensemble algorithm, we discuss different strategies for clustering predicted sites. A drawback of sorting predicted sites by their score is that sites from different motifs in a sequence can be mixed and clustered together, although it has an advantage of increasing the specificity of the final prediction. Alternatively, all predicted sites for a sequence can be placed on the sequence and votes can be cast to sequence positions occupied by predicted sites. Votes from a predicted site can reflect its score assigned by the component algorithm. Another idea is after grouping predicted sites by the score for each algorithm as it is done in the current implementation, each group can be corresponded to other overlapping groups (in terms of their location in sequence) from a different algorithm, but not by the score rank.

Weighting predicted sites is another important issue in an EMD algorithm. In the current implementation, all predicted sites have an equal weight of one, although as it is mentioned above, the significance scores assigned to them by each algorithm are implicitly counted in the clustering phase. Generally, a weight to a predicted site will originate from two sources, from the reliability of individual prediction indicated by the score and from the reliability of the algorithm itself, which can be measured by the overall prediction accuracy on a certain benchmark dataset. The weighting of sites can be considered in the voting phase in our implementation. Counting the two sources of weights, a vote for a predicted site may be revised to w_1b_*w_2a _instead of one, where w_1 _will be proportional to the assigned score to a particular site, b, and w_2 _will be proportional to the overall accuracy of the algorithm, a. One way of doing this is to develop reasonable formulae for w_1 _and w_2 _and see if the weights improve the overall results or not. The difficulty lies especially in determining weights for algorithms, w_2a_, because they depend on the combination of component algorithms in an EMD algorithm. As shown in Table 2, sometimes a combination of a relatively more accurate algorithm and a relatively less accurate one performs better than a combination of two average ones, so the resulting performance of an EMD algorithm depends on the compatibility of component algorithms, not necessarily to the accuracy of individual algorithms. Another way to change the strength of contribution of an algorithm is to change the number of runs.

An alternative way to find appropriate weights will be to use an optimization algorithm such as neural network or genetic algorithm. The weights assigned to component algorithms in an EMD algorithm, w_2a_, could be optimized. As for the weight assigned to individual site prediction, first a formula for w_1b _should be developed using several adjustable parameters, and those parameters will be optimized. An advantage of using an optimization technique is that the other parameters such as the number of runs and/or built-in parameters for each component algorithm may be able to be optimized at the same time. Again since the appropriate weights and the other parameters may totally depend on the combination of component algorithms, they should be optimized every time a different combination is tried.

### Diversity of predictions from component algorithms

To ensure that the predictions from component algorithms cover most target motifs, it is desirable that diverse predictions are generated from component algorithms. Diversified predictions would contribute in increasing the sensitivity of the final prediction. For stochastic algorithms, multiple runs usually naturally generate multiple different predictions. For deterministic algorithms like MEME and MDScan, we changed some parameters (named diversity parameters) to force them to generate different predictions. This idea of the diversity parameter is reasonable since the optimal parameter for a given input data set cannot be estimated in advance. However, a possible downside is that if the parameter is excessively changed, the quality of the predictions can be significantly deteriorated, resulting in a poor consensus building. Another possible way to diversify predictions is to feed different runs or algorithms with different subset data sampled from the original input sequences, which is yet to be explored.

## Conclusion

We have introduced an ensemble approach to the motif discovery problem. To the best of our knowledge, this is the first time that an ensemble approach is used in the motif discovery problem. By combining multiple predictions from multiple runs of one or more component algorithms, our ensemble algorithm showed good improvement in the sensitivity and specificity and thus, the overall accuracy over stand-alone component algorithms. The EMD algorithm is scalable in the sense that the EMD algorithms performed better, or at worst, equally compared to individual component algorithms. The improvement in the accuracy over the component algorithm is more significant for shorter input sequences. Considering the importance of the regulatory motif discovery in gene expression analysis and the poor performance of the current individual motif discovery algorithms, our EMD algorithms can be a very useful tool in the era of systems biology.

## Methods

### Benchmark data sets

The benchmark data set is generated from RegulonDB [38], which stores motif information of *E. coli *K12. Since this benchmark set is basically the same as the one used in our previous study [6], we only briefly describe it here.

Three types of data sets (Type A, B, and C) are prepared. Type A data sets are generated from the intergenic regions of *E. coli *genome. The intergenic region sequences are taken from the KEGG database [39]. This set A contains 62 motif groups and is called ECRDB62A. It has the following characteristics: the average number of sequences per motif group is 12; the average number of sites per sequence is 1.85; the average sequence length is 300 nt; the average site width is 22.83.

Types B and C data sets include sequences with symmetric margins on both sides of known sites. The difference between the sets B and C is that actual flanking sequences in the *E. coli *genome are used for margins for the set B, whereas artificial sequences are used for the type C.

Set B is termed ECRDB61B-X, where X denotes the margin size of the sequences. The length of the margins is changed from 20 to 800, so that the scalability of algorithms in terms of the input sequence length can be examined. Because margins are added on both sides of a site, the actual length of an input sequence is the total of the site width and two times the margin size. The number 61 denotes the number of motif groups available in this benchmark set. When a large margin size is used, it sometimes happened that an extended sequence includes another site of the same motif group, thus the two input sequences contain the same set of two sites of the same type. In such a case, one of the input sequences is removed, and if this procedure reduces the number of input sequence to one, the entire motif group is removed from the benchmark dataset. It also happened that an input sequence of a certain motif group contains another site of a different type when a large margin size is used. But since this case happens in a real situation, primarily we kept these sequences in the dataset. In such a case, because sites of a target motif are still abundant, we expect an algorithm is able to pick the target sites as one of the top K scoring motifs. We also observe that when the margin sizes are larger (*e.g*. >500 nt), some part of the sequences are located in the coding regions. However, as shown in the previous study [6], no significant influence has been observed of these variations on the prediction accuracy. In ECRDB62A and ECRDB61B-X dataset, there are input sequences which have multiple identical motifs (i.e. two AraC motifs on a sequence) on it. In those cases, positions of both motifs are considered to be correct. And the dataset is cleaned so that a certain motif position only occurs once in a motif group. Also a motif group does not contain different motifs multiple times, so that the motif discovery algorithms do not confuse by them.

In addition to the type A and B sets, we have prepared the type C set, where artificially shuffled sequences are used for margins on both sides of known sites. This dataset is termed ECRDB61C-X. X represents the length of the margin sequence. The same set of the known sites are used as in ECRDB61B data set. Three different margin sizes, 100, 200, and 400 nt. are used. The margin sequences are artificially shuffled, while preserving the di-mer nucleotide frequency of intergenic regions of the *E. coli *genome. The motivation of using the type C set is to evaluate the performance of the algorithms on sequences which surely contain only one target regulatory site in a sequence. The benchmark data sets are available at our web site [40].

### Ensemble motif discovery algorithm

The Ensemble Motif Discovery (EMD) algorithm represents a family of motif discovery algorithms that combine multiple results from individual component algorithms. In this study, we primarily used five component algorithms which are described in the next section. A certain ensemble algorithm can be uniquely identified by the set of component algorithms used, the number of times each component algorithm is run (R_i_, i denotes for a certain component algorithm), the parameter set used to run each component algorithm (P_i_), and the number of top scoring motifs considered from each run (T_i_). Thus, an EMD algorithm can be specified as EMD(-CA_i_-R_i_-P_i_-T_i_)_M_, where CA_i _denotes a certain component algorithm with the conditions, R_i_, P_i_, T_i _(i goes from 1 to *M*, *M *is the number of different component algorithms combined).

More generally, P_i _and T_i _can be changed for each different run of the algorithm i, which can introduce further variations to the EMD algorithms. However, since it is not possible to explore every possible condition, some of the parameters are set to be the same. The number of motifs to be reported from individual runs is set to five for all runs of all the component algorithms (T_i _= 5). The number of runs (R_i_) is set to 20 if not specified otherwise for all component algorithms. For a single component algorithm, the same parameter set is used for all the runs. For deterministic algorithms, however, the parameter set is changed for each run because otherwise they produce identical results. For a deterministic algorithm, ten different values are prepared for a tuning parameter (we call it a diversity parameter), one of which is selected randomly for each run. Since we don't change R_i _and T_i _for each different algorithm i, and the parameter set P_i _is set to be same in all the runs of the algorithm i, the notation of an EMD algorithm can be simplified to be EMD(-CA_i_)_M_. Specifically, we denote EMD-X (X = 1~5) as the set of all the possible combinations of X component algorithms.

### Algorithm of the EMD

Suppose an EMD algorithm combines *M *component algorithms, A_i_, (i = 1..M). The EMD algorithm predicts motifs in a set of *N *sequences, S_i_, (i = 1..N). Here a *motif *is defined as a set of local regions (=*sites*) in input sequences which are detected to be similar to each other. In the other words, one or sometimes more *sites *are predicted in each input sequence, and all of the sites in an input sequence set form a *motif*. The EMD algorithm has five steps to generate its final prediction: collecting, grouping, voting, smoothing, and extracting. The overview of the algorithm is provided in Figure 1.

#### (1) Collecting

Each algorithm A_i _runs *R *times against an input dataset of *N *sequences and reports top *K *scoring motifs for each run. For a motif, a component algorithm A_i _usually detects one site per input sequence by a single run, resulting the total of R*K predicted sites in a sequence. If an algorithm A_i _reports more than *K *motifs, only the top *K *scoring motifs are considered. Oppositely, if less than *K *motifs are predicted by an algorithm, all of the motifs are considered. Also since sometimes an algorithm picks more than one site or no sites in a sequence, the total number of predicted sites in a sequences can be more or less than R*K. Now for each combination of an input sequence and a component algorithm, all the predicted sites are collected. Figure 1 illustrates the collecting phase of sites in the input sequence number 1 from all the algorithms, A_1 _to A_M_.

#### (2) Grouping

From the collecting phase above, for an input sequence S_i _we have about R*K predicted sites from each of the algorithm A_i_. In the grouping phase, first, all the predicted sites in an input sequence S_i _by a certain algorithm A_i _are sorted by the algorithm's major statistical score. Then the predicted sites are divided into *K *groups by the sorted score, with each of the groups having an equal number of predicted sites. Because usually an input sequence has R*K sites, most of the groups have *R *sites.

Then, the groups of the same score rank across the results by *M *different algorithms are joined together. This results in *K *groups of predicted sites from all predictions made by all algorithms.

The reason for employing this sorting step is to take the score assigned to predicted sites by the algorithm into account. We have observed from prediction results of single component algorithms that the correct site position is frequently predicted within the top couple of score ranks most of the time. Therefore, a local region predicted as the target site consistently in every run by the algorithm within the top scores can be considered to be more reliable.

Basically, each of the *K *groups will finally produce a predicted site. Thus in total EMD outputs *K *predicted sites for an input sequence. But if the average number of predicted sites, *B*_*i*_, for a given input sequence S_i_, is more than one, there is an option to output *B*_*i *_sites for the sequence S_i _from each group. The subsequent steps of voting, smoothing, and extracting steps explain how EMD construct final predictions from the collected sites in each group.

#### (3) Voting

For each of the *K *predicted site groups in a sequence S_i_, all the predicted sites in a group are placed on the sequence. Then for each position p in a sequence, the number of times the position p is included, or votes for the position p, *V*_*p*_, in the predicted sites is counted. Figure 1 shows the number of votes *V*_*p *_along the sequence position p for the site group 1.

#### (4) Smoothing

The vote *V*_*p *_along an input sequence is smoothed using a sliding window of a width of *W*_*s*_, which is half of the specified motif width *W *(*W *is a user specified parameter, the default value is 15 and W_s _is 8). The sliding window starts from the left most position of the sequence, and the sum of the votes in the window, Vps
 MathType@MTEF@5@5@+=feaafiart1ev1aaatCvAUfKttLearuWrP9MDH5MBPbIqV92AaeXatLxBI9gBaebbnrfifHhDYfgasaacH8akY=wiFfYdH8Gipec8Eeeu0xXdbba9frFj0=OqFfea0dXdd9vqai=hGuQ8kuc9pgc9s8qqaq=dirpe0xb9q8qiLsFr0=vr0=vr0dc8meaabaqaciaacaGaaeqabaqabeGadaaakeaacqWGwbGvdaqhaaWcbaGaemiCaahabaGaem4Camhaaaaa@30E6@, is placed in the center position *p *of the window. In the case that *W*_*s *_is even, the smoothed score, Vps
 MathType@MTEF@5@5@+=feaafiart1ev1aaatCvAUfKttLearuWrP9MDH5MBPbIqV92AaeXatLxBI9gBaebbnrfifHhDYfgasaacH8akY=wiFfYdH8Gipec8Eeeu0xXdbba9frFj0=OqFfea0dXdd9vqai=hGuQ8kuc9pgc9s8qqaq=dirpe0xb9q8qiLsFr0=vr0=vr0dc8meaabaqaciaacaGaaeqabaqabeGadaaakeaacqWGwbGvdaqhaaWcbaGaemiCaahabaGaem4Camhaaaaa@30E6@, is placed at the *q*-th position in the window, where *q *= (*W*_*s*_/2)+1.

#### (5) Extracting final prediction of sites

The final stage is to pick up the top local peak (or the top *B*_*i *_local peaks) in the smoothed voting curve, Vps
 MathType@MTEF@5@5@+=feaafiart1ev1aaatCvAUfKttLearuWrP9MDH5MBPbIqV92AaeXatLxBI9gBaebbnrfifHhDYfgasaacH8akY=wiFfYdH8Gipec8Eeeu0xXdbba9frFj0=OqFfea0dXdd9vqai=hGuQ8kuc9pgc9s8qqaq=dirpe0xb9q8qiLsFr0=vr0=vr0dc8meaabaqaciaacaGaaeqabaqabeGadaaakeaacqWGwbGvdaqhaaWcbaGaemiCaahabaGaem4Camhaaaaa@30E6@. Then a window of the length *W *is placed as the final prediction of the site, with the center of the window positioned at the peak.

The smoothing and the extracting phases are aimed to decide the final site prediction by majority votes. Although it may be possible that minority votes for different motifs are not selected as one of the final predictions, but this procedure will be superior in picking up sites which are consistently predicted with a high score rank. Alternative ways to combine different predictions are discussed in Discussion.

### Component algorithms

There are several factors that need to be considered to develop an ensemble algorithm. First, we have to identify whether a component algorithm is a stochastic or deterministic algorithm. In an ensemble algorithm, the component algorithms are usually run multiple times and all the results are combined together. We need a way to control the proportion of predictions from different algorithms to avoid any bias. For deterministic component algorithms, such as MDScan [32] and MEME [33], multiple runs generate identical predictions, which will strongly bias the final combined result. To address this problem, we introduced the diversity parameter(s), which is defined as one or more parameters of an algorithm that one can tune to generate different predictions. In this study, only one diversity parameter is chosen for the deterministic algorithms, namely, MEME and MDScan.

Five motif discovery programs, namely, AlignACE [30], MEME [33], BioProspector [31], MDScan [32], and MotifSampler [34] are selected as the component algorithms for composing the ensemble algorithms. These algorithms only use DNA sequence information as input to identify the regulatory motifs. These algorithms are selected because of their wide use and being ready for download from the internet, allowing us to do large scale local runs. Below we describe the parameter setting of each algorithm. A random algorithm is also introduced to evaluate the statistical significance of the prediction accuracy of the ensemble algorithm.

One difficulty in testing the performance of an algorithm is to set optimal parameters. Here most of the parameters for the component algorithms are set as default values except those which can be easily estimated from general biology knowledge, as we did in the previous study [6]. For example, we have chosen 15 as the expected motif width for the component algorithms (except for MEME, which can adjust motif width by itself), because 15 is the approximate average between the default value of the algorithms and the average motif width in ECRDB62A, which is 21. The reason why we used default parameters (except for the few parameters mentioned below) for the component algorithms is that it is infeasible to try all the possible combinations of parameters of multiple component algorithms, and the default setting for an algorithm should work reasonably well in most of the cases, because it is set up by the authors of the algorithm. The parameter set used for each component algorithms can be found at our web site [40]. This is the same parameter set used in the previous study [6].

### AlignACE

AlignACE [30] is a stochastic motif discovery program based on the widely adopted Gibbs Sampling method [11]. Running parameters for AlignACE were set as the default except for the background fractional GC content gcback set to 0.5, which is calculated from the whole *E. coli *genome. The expected motif width was set to 15. The major statistical score in AlignACE is the MAP score, being the larger is better.

### BioProspector

BioProspector [31] is another variant of the Gibbs Sampling algorithm, which has fifteen parameters to fine-tune its prediction behavior. We used the default values for most of these parameters except for: the motif width, which was set to 15; the number of top motifs to report, which was set to 5. The background frequency model was generated using the whole *E. coli *genome and the third order Markov model was used. The order of the background model and subsequent ones for MDScan, MotifSampler and MEME was determined based on our previous benchmark study of these component algorithms [6]. BioProspector uses a maximum a posterior (MAP) score to evaluate candidate motifs.

### MDScan

MDScan [32] is an enumerative deterministic greedy algorithm. Among its ten parameters, we only specified the following parameters. The motif width was set to 15. The background frequency model was generated using the whole *E. coli *genome and the third order Markov model was used. MDScan uses a maximum a posterior (MAP) score to evaluate candidate motifs. We chose the -s parameter < the number of top motifs to scan and refine > as the diversity parameter to generate different predictions for multiple runs. It ranged from 10 to 100 with a step size of 10.

### MEME

MEME (Multiple Expectation Maximization Estimation) [33] is a deterministic algorithm based on the expectation maximization (EM) technique. It is the only algorithm in this evaluation that does not require a motif width parameter, because MEME can estimate by itself. MEME has 28 parameters. We set the maximum dataset size in characters to one million, the maximum running time to 3600 CPU seconds, the maximal number of motifs to find to five, and the minimum number of sites for each motif to one. The third order Markov model was used for the background frequency model. Default values were used for all the other parameters. We chose the -maxw <maximum motif width> as the diversity parameter, ranging from 10 to 19, with the step size of 1.

### MotifSampler

MotifSampler [34] is another motif discovery program based on Gibbs sampling. MotifSampler has seven major parameters. We made the following adjustments to the default parameter values. We searched five different motifs of a width of fifteen. The number of repeating runs was set to five. The background frequency model was generated using the intergenic region sequences of all *E. coli *genome and the third order Markov model was used. We used the consensus score as the statistical measure for the quality of the predicted motifs.

### The multi-restart algorithm

One interesting question for ensemble algorithms is whether the performance improvement of the ensemble algorithm is due to more number of runs or to synergetic effect of multiple runs of multiple algorithms. We developed a multi-restart algorithm (RS) and compared its performance against that of ensemble algorithms. The basic idea of RS algorithm is to run a given algorithm multiple times and use the highest scored predictions as the final results. The multi-restart algorithm works as follows:

1) Run the component algorithm for *R *times, with each run reporting top *K *motifs.

2) Collect all the predicted motifs and sort them by the major statistical score of the algorithm.

3) Report the top *K *motifs among all the sorted motifs as the final prediction.

### The random algorithm

In a random motif algorithm, a certain number of sites are randomly picked up as predictions of sites. The number of sites picked up is decided for each input sequence as follows: First, conducted 10 runs of AlignACE, BioProspector, MotifSampler and one run of MEME to get the minimum (nSiteMin) and the maximum number (nSiteMax) of predicted sites. Then, the number of sites to be predicted is randomly chosen between nSiteMin and nSiteMax. The random algorithm is run 1000 times, and the average performance is reported.

### Measure of prediction accuracy

We use two levels of performance criteria: nucleotide and site levels to measure the prediction accuracy. A more detailed description is given in the previous study [6]. The nucleotide level accuracy measures include the performance coefficient (nPC), the sensitivity (nSn) and the specificity (nSp). The site level accuracy measures include the site level performance coefficient (sPC), the sensitivity (sSn) and the specificity (sSp). As described above, an EMD algorithm reports K (or sometimes less) motifs for a given input dataset. In this study, we evaluated the accuracy of the best prediction out of the K motifs [6].

### Nucleotide level accuracy

First, for each target site with overlapping predicted sites in an input sequence, we define the following values to calculate the accuracy metrics at the nucleotide level: nTP (true positive), the number of target site positions predicted as site positions; nTN (true negative), the number of non-site positions predicted as non-site positions; nFP (false positive), the number of non-site positions predicted as site positions; nFN (false negative), the number of target site positions predicted as non-site positions.

The nucleotide level performance coefficient (nPC), sensitivity (nSn) and specificity (nSp) for a pair of target/predicted sites is defined as:

nPC=nTPnTP+nFP+nFN,nSn=nTPnTP+nFN,nSp=nTPnTP+nFP     (1)
 MathType@MTEF@5@5@+=feaafiart1ev1aaatCvAUfKttLearuWrP9MDH5MBPbIqV92AaeXatLxBI9gBaebbnrfifHhDYfgasaacH8akY=wiFfYdH8Gipec8Eeeu0xXdbba9frFj0=OqFfea0dXdd9vqai=hGuQ8kuc9pgc9s8qqaq=dirpe0xb9q8qiLsFr0=vr0=vr0dc8meaabaqaciaacaGaaeqabaqabeGadaaakeaacqWGUbGBcqWGqbaucqWGdbWqcqGH9aqpdaWcaaqaaiabd6gaUjabdsfaujabdcfaqbqaaiabd6gaUjabdsfaujabdcfaqjabgUcaRiabd6gaUjabdAeagjabdcfaqjabgUcaRiabd6gaUjabdAeagjabd6eaobaacqGGSaalcqWGUbGBcqWGtbWudaWgaaWcbaGaemOBa4gabeaakiabg2da9maalaaabaGaemOBa4MaemivaqLaemiuaafabaGaemOBa4MaemivaqLaemiuaaLaey4kaSIaemOBa4MaemOrayKaemOta4eaaiabcYcaSiabd6gaUjabdofatnaaBaaaleaacqWGWbaCaeqaaOGaeyypa0ZaaSaaaeaacqWGUbGBcqWGubavcqWGqbauaeaacqWGUbGBcqWGubavcqWGqbaucqGHRaWkcqWGUbGBcqWGgbGrcqWGqbauaaGaaCzcaiaaxMaadaqadiqaaiabigdaXaGaayjkaiaawMcaaaaa@69F2@

In addition to the accuracy score for target sites with overlapping predictions, we need to address the cases of targeted sites which do not overlap with any predicted sites or predicted sites without overlapping any targeted sites. We define the number of non-overlapping target and predicted site pairs as the larger number among MT and MP, where MT denotes for the number of missing targeted sites and MP denotes for the number of wrong predictions. The accuracy scores of these non-overlapping pairs are set to zero. This definition will penalize algorithms that report either too many or too few site predictions. Based on the scores defined for the site pairs, the accuracy scores of a motif discovery program are calculated as:

1#_motif groups∑motif groups1#_sequences∑sequences1#_site pairs∑site pairsnPC(or nSp or nSn)     (2)
 MathType@MTEF@5@5@+=feaafiart1ev1aaatCvAUfKttLearuWrP9MDH5MBPbIqV92AaeXatLxBI9gBaebbnrfifHhDYfgasaacH8akY=wiFfYdH8Gipec8Eeeu0xXdbba9frFj0=OqFfea0dXdd9vqai=hGuQ8kuc9pgc9s8qqaq=dirpe0xb9q8qiLsFr0=vr0=vr0dc8meaabaqaciaacaGaaeqabaqabeGadaaakeaadaWcaaqaaiabigdaXaqaaiabcocaJiabc+faFjabd2gaTjabd+gaVjabdsha0jabdMgaPjabdAgaMjabbccaGiabdEgaNjabdkhaYjabd+gaVjabdwha1jabdchaWjabdohaZbaadaaeqbqaamaalaaabaGaeGymaedabaGaei4iamIaei4xa8Laem4CamNaemyzauMaemyCaeNaemyDauNaemyzauMaemOBa4Maem4yamMaemyzauMaem4CamhaaaWcbaGaemyBa0Maem4Ba8MaemiDaqNaemyAaKMaemOzayMaeeiiaaIaem4zaCMaemOCaiNaem4Ba8MaemyDauNaemiCaaNaem4CamhabeqdcqGHris5aOWaaabuaeaadaWcaaqaaiabigdaXaqaaiabcocaJiabc+faFjabdohaZjabdMgaPjabdsha0jabdwgaLjabbccaGiabdchaWjabdggaHjabdMgaPjabdkhaYjabdohaZbaaaSqaaiabdohaZjabdwgaLjabdghaXjabdwha1jabdwgaLjabd6gaUjabdogaJjabdwgaLjabdohaZbqab0GaeyyeIuoakmaaqafabaGaemOBa4MaemiuaaLaem4qamKaeiikaGIaem4Ba8MaemOCaiNaeeiiaaIaemOBa4Maem4uamLaemiCaaNaeeiiaaIaem4Ba8MaemOCaiNaeeiiaaIaemOBa4Maem4uamLaemOBa4MaeiykaKcaleaacqWGZbWCcqWGPbqAcqWG0baDcqWGLbqzcqqGGaaicqWGWbaCcqWGHbqycqWGPbqAcqWGYbGCcqWGZbWCaeqaniabggHiLdGccaWLjaGaaCzcamaabmGabaGaeGOmaidacaGLOaGaayzkaaaaaa@A914@

Thus, the score is first averaged over all site pairs in a sequence, followed by averaging over all sequences in a motif group, and finally averaging over all the motif groups. Note that we allow multiple sites on a sequence as targeted sites.

### Site level accuracy

The site level accuracy indicates if predicted sites overlap with true sites by one or more nucleotide position. We define, sTP, sFP, and sFN as follows: sTP, the number of predicted sites which overlaps with the true sites by at least one nt; sFP, the number of predicted sites which have no overlaps with the true sites; sFN, the number of true sites that have no overlaps with any predicted sites.

For each input sequence, we define the site level performance coefficient (sPC), sensitivity (sSn), and specificity (sSp) in the following way:

sPC=sTPsTP+sFP+sFN,sSn=sTPsTP+sFN,sSp=sTPsTP+sFP     (3)
 MathType@MTEF@5@5@+=feaafiart1ev1aaatCvAUfKttLearuWrP9MDH5MBPbIqV92AaeXatLxBI9gBaebbnrfifHhDYfgasaacH8akY=wiFfYdH8Gipec8Eeeu0xXdbba9frFj0=OqFfea0dXdd9vqai=hGuQ8kuc9pgc9s8qqaq=dirpe0xb9q8qiLsFr0=vr0=vr0dc8meaabaqaciaacaGaaeqabaqabeGadaaakeaacqWGZbWCcqWGqbaucqWGdbWqcqGH9aqpdaWcaaqaaiabdohaZjabdsfaujabdcfaqbqaaiabdohaZjabdsfaujabdcfaqjabgUcaRiabdohaZjabdAeagjabdcfaqjabgUcaRiabdohaZjabdAeagjabd6eaobaacqGGSaalcqWGZbWCcqWGtbWucqWGUbGBcqGH9aqpdaWcaaqaaiabdohaZjabdsfaujabdcfaqbqaaiabdohaZjabdsfaujabdcfaqjabgUcaRiabdohaZjabdAeagjabd6eaobaacqGGSaalcqWGZbWCcqWGtbWucqWGWbaCcqGH9aqpdaWcaaqaaiabdohaZjabdsfaujabdcfaqbqaaiabdohaZjabdsfaujabdcfaqjabgUcaRiabdohaZjabdAeagjabdcfaqbaacaWLjaGaaCzcamaabmGabaGaeG4mamdacaGLOaGaayzkaaaaaa@6A0C@

The site level accuracy score for an input sequence set (or a motif group) is the average of the score over all the sequences. The site level accuracy score of the entire benchmark data set is the average of the scores for all input sequence sets.

## Authors' contributions

JH designed and implemented the algorithm, carried out the initial benchmark study and drafted the manuscript. YDY carried out additional benchmark study required to revise the manuscript. DK conceived of the study, participated in its coordination, and wrote the manuscript. All authors read and approved the final manuscript.

## Figures and Tables

**Table 1:** The prediction accuracy tested on ECRDB62A set.

Algorithm	nPC	nSn	nSp	sPC	sSn	sSp
BP-MD ^a)^	0.183	0.215	0.280	0.303	0.428	0.407
**AL-BP-MD**	**0.213**	**0.262**	**0.296**	**0.324**	**0.456**	**0.437**
AL-BP-MD-MS	0.209	0.255	0.293	0.321	0.423	0.446
AL-BP-MD-ME-MS	0.197	0.238	0.286	0.316	0.438	0.437

AlignACE	0.141	0.218	0.171	0.264	0.351	0.396
**BioProspector**	**0.174**	**0.205**	**0.268**	0.287	0.415	0.369
MDScan	0.146	0.174	0.223	0.244	0.345	0.349
**MEME**	0.160	0.260	0.190	**0.300 **^d)^	**0.440**	**0.430**
**MotifSampler**	0.150	0.180	0.230	**0.300**	**0.320**	**0.490**

RS-AL ^b)^	0.139	0.204	0.166	0.229	0.329	0.341
**RS-BP**	**0.150**	**0.178**	**0.231**	**0.262**	**0.390**	**0.350**
RS-MD	0.107	0.125	0.169	0.170	0.254	0.271
RS-ME	0.133	0.162	0.203	0.213	0.418	0.282
RS-MS	0.127	0.148	0.187	0.235	0.260	0.384

Random ^c)^	0.050	0.061	0.083	0.100	0.161	0.146

a) The best algorithm among EMD-X (X = 2~5) are compared with component algorithms, b) the multi-restart algorithms, and c) the random algorithms. The best performances in terms of nPC or sPC among algorithms of a same category are highlighted in bold. d) Both MEME and MotifSampler are highlighted because they have the same performance in terms of sPC.

**Table 2:** Comparison of nucleotide level prediction accuracy (nPC) of EMD algorithms with different combinations of component algorithms ^a)^.

Algorithm	nPC	Algorithm	nPC	Algorithm	nPC
AL	0.178	AL-BP	0.213	AL-BP-MD	0.239
BP	0.201	AL-ME	0.203	AL-BP-ME	0.225
**MD**	**0.228**	AL-MS	0.208	AL-BP-MS	0.213
ME	0.199	BP-MD	0.241	**AL-MD-ME**	**0.250**
MS	0.206	BP-ME	0.214	AL-MD-MS	0.241
Average	0.202	ME-MS	0.220	AL-ME-MS	0.220
				
**AL-BP-MD-ME**	**0.254**	BP-MS	0.207	BP-MD-ME	0.243
AL-BP-MD-MS	0.244	MD-ME	0.235	BP-MD-MS	0.249
AL-BP-ME-MS	0.232	AL-MD	0.240	BP-ME-MS	0.223
AL-MD-ME-MS	0.252	**MD-MS**	**0.250**	MD-ME-MS	0.241
BP-MD-ME-MS	0.248	Average	0.223	Average	0.234
		
Average	0.246			AL-BP-MD-ME-MS	**0.254**

a) Tested on ECRDB61B-200 data set. b) The best EMD algorithms among those of a given number of component algorithms are highlighted in bold.

**Table 3:** The performance (nPC) of EMD-2 algorithms with respect to the number of runs of its component algorithms ^a)^.

Algorithm\No. of Runs	5	10	15	20
AL-BP	**0.219**	0.216	**0.219**	0.213
AL-ME	0.231	0.231	**0.241**	0.240
AL-MS	0.182	**0.208**	0.203	0.203
BP-MD	0.190	0.206	0.197	**0.208**
BP-ME	0.240	**0.242**	0.238	0.241
ME-MS	0.210	**0.216**	0.214	0.213
BP-MS	**0.208**	0.207	0.207	0.207
MD-ME	0.222	0.233	0.227	**0.235**
AL-MD	0.236	0.236	0.236	**0.250**
MD-MS	0.195	**0.225**	0.220	0.220
Average	0.214	0.222	0.220	**0.223**

a) ECRDB61B-200 (margin size of 200) data set is used.

b) The nPC value is in bold if it is the best performance among tested for that EMD algorithm.

**Table 4:** The performance of AL-BP-MD-ME-MS on a dataset of shuffled margin sequences ^a)^.

	nPC			nSn			nSp		
Margin size (nt)	100	200	400	100	200	400	100	200	400
Original set ^b)^	0.288	0.254	0.197	0.328	0.292	0.234	0.416	0.360	0.270
Shuffled set ^c)^	0.317	0.255	0.187	0.340	0.275	0.201	0.481	0.375	0.266

a) To the both sides of known sites in the ECRDB61B data set, artificially shuffled sequences with the size of 100, 200, and 400 nt are attached. The statistics of the di-mer nucleotide frequency used to generate the shuffled margin sequences are taken from intergenic regions of the *E. coli *genome.

b) The performance on the original ECRDB61B-100, 200, 400 set.

c) The performance on the data set with shuffled margin sequences.
